# The Discursive Configuration of the Therapeutic Community for Substance Users: Positioning and Ethnopsychological Processes Concerning Entry

**DOI:** 10.3390/bs14100951

**Published:** 2024-10-15

**Authors:** Antonio Iudici, Tobia Berardelli, Davide Fenini, Emiliano Subissi, Jessica Neri

**Affiliations:** 1 Department of Philosophy, Education, Sociology and Applied Psychology of Padua (FISPPA), University of Padua, 35139 Padua, Italy; t.berardelli@scuolainterazionista.it (T.B.); j.neri@scuolainterazionista.it (J.N.); 2 Institute of Psychology and Psychotherapy, Scuola Interazionista, 35100 Padua, Italy; d.fenini@scuolainterazionista.it (D.F.); e.subissi@scuolainterazionista.it (E.S.)

**Keywords:** users’ perspective, discourse analysis, interactionism, residential community, health promotion

## Abstract

Therapeutic communities face high drop-out rates and general distrust of their effectiveness among substance users. Actively involving users early in treatment promotes greater compliance with the treatment and is predictive of better outcomes. However, users often occupy a passive and subordinate role, exacerbated by the lack of research that explores their perspectives, beliefs, and experiences. This study examined the discourses of 57 consumers who were part of a community for less than 15 days, investigating the meanings attributed to service entry and treatment. A protocol of four written open-ended questions was employed and analysed through discourse analysis and positioning theory. The results indicate that participants configure the community as a place symbolically and spatially distinct from the rest of the world, where they isolate themselves to seek support during times of extreme difficulty. However, what they are seeking is a solution to acute distress caused by substance use, intertwined with social, economic, and relational issues. The concept of treatment is built on the image of the substance user as an individual making a weak request for help, attributing the problem solely to drugs and exhibiting reduced agency in addressing their issues. The collected texts provide a better understanding of the experiences of new users, highlighting the importance of co-constructing personalised projects that empower consumers to feel actively involved in their own change, exploring their theories and definitions of self to structure pathways based strictly on their needs.

## 1. Introduction

Interventions related to substance abuse are complex and involve various disciplinary fields, including medical–pharmacological, psychological, social, and occasionally, psychiatric [1]. The study of the physiological effects of psychoactive substance consumption has expanded the tools and intervention possibilities from a biochemical standpoint. Such possibilities include the implementation of pharmacological support for detoxification, tolerance, and dependence [2], as well as the study of comorbidity situations when substance use is associated with other disorders that exacerbate the course of both [3].

The results achieved in this field have shifted the research focus on substance use towards quantitative data [4,5], with a prevalence of approaches that reduce mental conditions of the brain’s physical structure and functioning [6,7,8,9], favouring a reductionist conception of the overlap between substance use and the neurophysiological effects of consumption. Indeed, individuals who use substances have often been studied as ill patients before being considered as persons [10]. Consequently, the focus of studies and interventions has been on ‘substance use’ as a pathology and ‘drug users’ as individuals with disorders. Psychological research has predominantly sought elements of distress or cognitive factors that could explain relapses and therapeutic failures [11]. Across these research domains, there has been an overall interest in addressing people seeking treatment for substance use primarily as passive subjects with a lack of self-determination and diminished cognitive capacities [12]. This approach has led to the reduced involvement of individuals who use substances in analysing their responsibilities, experiences, beliefs, and methods of addressing social reintegration and treatment [4,8,13,14].

Conversely, some authors argue that understanding how consumption occurs and replicates necessitates studying the cultural and social narratives present in specific contexts, delving into how these narratives are practised and interactively re-signified [15,16]. For instance, Copes’ meta-analysis [17] illustrates how substance use places individuals in a position of extreme identity vulnerability, emphasising the need to focus on symbolic and existential aspects, as well as biomedical ones. The literature review revealed that individuals with substance use disorders approached treatment and residential communities in a subordinate role, predominantly delegating their rehabilitative pathway to the professionals within these facilities [4,18,19].

This perception of consumption as an inability to cope with use suggests that individuals are at the mercy of drugs for life [20]. Some authors argue that this passivity and sense of incapacity may partly explain the significant level of the inefficacy or discontinuation of rehabilitative treatment [21,22]. Indeed, it has been found that 50% of individuals admitted to communities did not complete the first 10 months of a programme, and 45% of consumers remained unaware of the available services [23,24].

Several studies have highlighted how the intake phase is particularly crucial for the programme’s trajectory [5]. Specifically, this phase is significant for forming the so-called ‘therapeutic alliance’ [25] and for defining a solid, enduring path. Some authors argue that treatment is more effective based on the duration of stay [5] and user involvement [26]. Others have underscored the importance of establishing a co-operative relationship in treatment [27], involving the consumer and their expectations [28,29]. Some authors emphasise the importance of collaboration between service providers and users in the initial stages of the pathway, particularly the need to tailor treatment to the user’s characteristics [30,31,32]. Some authors [24,33,34] argue that the way individuals who use substances access services is much more varied than the stereotypical conceptions of ‘motivation’ and ‘personal choice’. This study aims to analyse the narratives and interactive modalities involved in entering the therapeutic community (TC) and utilising interventions by capturing the discourses that lead substance users to seek help in communities and understanding their approach to treatment. A TC is a service in which individuals with addictive (and other) problems live together in an organised and structured manner to promote change and facilitate a drug-free life in society [35].

Based on the research and considerations presented, this study begins with specific questions: What is the basis for substance users to request help? What kind of assistance do they anticipate from the community?

This study, therefore, focuses on the moment of access to residential services, delving into how consumers assign meaning to the service itself. This investigation can provide a deeper understanding of the discursive construction of therapeutic communities and the identities of individuals who use substances, specifically the socio-cultural and contextual elements that contribute to shaping the phenomenon and its potential trajectories of change. Analysing the interactive dynamics of accessing the residential community can illuminate the complexities of the help-seeking process. It can uncover barriers or facilitators of engaging with services, as well as highlighting opportunities for improving the delivery of care.

## 2. Materials and Methods

### 2.1. Theoretical Background

This study adopts the interactionist paradigm as its theoretical framework [36,37,38,39]. In this paradigm, the individual is conceptualised as an active agent constantly negotiating their reality based on the meanings they attribute to it, which are generated interactively within socio-cultural matrices [40,41,42,43].

The self is seen as having a dialogical character [44,45], positioning it as an intersection between multiple levels. Identity is thus viewed as a dialogical process that allows for the interaction of intrapersonal (self-attribution) and interpersonal (other attribution) elements within the cultural matrix of reference, converging into an organising structure of self-knowledge [46,47,48]. Therefore, attention is placed on analysing discursive and interactive processes that contribute to the configuration of the phenomena under investigation [41,49]. Regarding this, entry into a community, like any institutionalised or therapeutic context, is considered significant when analysing the discursive processes used by those who populate it [50,51] and their pragmatic implications.

### 2.2. Aims

This study aims to investigate how individuals who use legal and illegal psychoactive substances shape the residential community and their activities during the entry phase into a TC. Specifically, considering the potential biographical transition represented by entering into such a service, attention has been focused on the following areas of investigation:

- Analysis of the discursive configuration regarding the entry of substance users into the service;
- Analysis of the discursive configuration regarding expectations and activities within the community.

These areas of investigation allow for an in-depth exploration of the theories, beliefs, and expectations of individuals entering TCs regarding the entry and utilisation of interventions promoted within these contexts.

### 2.3. Participants

The research was conducted in northern Italy, specifically in the regions of Veneto and Lombardia. These regions were chosen because they host the highest number of therapeutic communities in Italy. The study included 57 individuals who had accessed four therapeutic–rehabilitative residential communities for drug addiction within the previous 15 days.

For participant recruitment, an initial mapping of local services was conducted based on the provision of programmes for managing and treating substance-related issues. These facilities were contacted, and the research objectives were shared with the responsible personnel. Potential participants were then approached and invited to collaborate, with the research objectives and methods explained to them. Regarding entry requirements, all participants were poly-consumers, predominantly alcohol and drug users. All participants had previously attended at least one other community in their lives. This requirement was established to detect established theories about therapeutic communities. The questionnaire was shared with them shortly before entering the new community. Individuals who used only one substance or were entering a community for the first time were excluded from the research. Participants were divided into (a) substance users who had a regular entry, that is, based on a referral from the Services for First Integration (Ser.D), and (b) individuals using substances accessing the community as an alternative to prison. Under Italian law [52], some detained persons can follow a community-based treatment pathway (alternative measure programme). Each participant was provided with a module to give their informed consent. Each participant was asked to complete a written questionnaire. Our study has been approved by the University of Padua’s Ethics Committee for Research (protocol code: 3653).

Table 1 presents the demographic data of the participants. It is noteworthy that most participants are male, and the average age is 37.7 years, which aligns with the national average data reported by the Department of Anti-Drug Policies [53].

### 2.4. Data Collection

According to the theoretical background, a qualitative approach was adopted to understand the complexity of the experiences provided by the participants, limiting the risk of reducing them to quantitative categories. The research is understood as a relational, collaborative practice and dialogical process [54,55], with the object of analysis considered co-constructed by the researchers and the interlocutors.

The research instrument consists of a written protocol of six open-ended questions (Table 2).

The questions were designed to elicit responses adhering to specific themes while allowing for a breadth of content and form [56]. The choice of open-ended questions was crucial within the qualitative research paradigm [57,58], particularly in collecting written text [59,60,61,62,63]. The question prompts were constructed concerning the research objective and its unique areas of investigation to delineate the scope of potential discourses. At the same time, the open-ended questions allowed ample room for participants to freely express themselves and manage their responses and relationships with the researcher present.

The questions were presented to participants individually, clarifying the methods and objectives of the research, ensuring anonymity, and assuring that the produced texts would not be disclosed to service personnel. The researcher was available to clarify any questions during the completion process. Responses were provided in written form autonomously, taking between 25 and 60 min.

The responses were transcribed verbatim and reviewed with the participants before delivery to ensure understanding of the written text [64,65]. In two cases of limited literacy, the participants dictated their answers to the researcher, who transcribed and read the answers immediately. The completion occurred within 15 days of entering the community. The texts were then translated into English.

### 2.5. Data Analysis

The texts were analysed using discourse analysis [66,67]. This method aims to capture the narratives (or discursive positioning) present in the written text, analysing simple content and the discursive processes through which specific theories and ideologies are perpetuated in the text. The framework of positioning [68,69,70] was utilised to underscore the dynamic aspects through which the discursive shaping of identity occurred, such as that of substance users.

Adopting a particular positioning allows one to perceive the world from that standpoint, interpreting it through the lens of the relevant images, metaphors, and concepts within the discursive frameworks in which one is situated [68]. Moreover, positioning manifests the illocutionary and intentional force of the speaker, utilising language to position oneself and others within a framework of rights and obligations, potentially affecting certain outcomes [71]. Special attention was devoted to analysing the socio-cultural and ethnopsychological elements of the narratives, as they are significant and central in the discursive construction of the investigated reality.

The analysis of discourse and positioning made it possible to explore how participants articulated their narratives and positions concerning the area of investigation: entry into the community. The analysis of discursive processes enables the identification of the most pertinent discourses and positions, elucidating their practical implications within the life stories and pathways of intervention for the participants.

The analytical procedure involved a comprehensive examination of the texts written by the participants, conducted separately for the two primary thematic areas explored in this study. This re-evaluation process occurred at various points in time and involved the collaboration of three different researchers to pinpoint any discrepancies in the analysis.

Upon identifying the fundamental discursive patterns related to positioning, we organised the findings into groups to enhance clarity and facilitate the interpretation of the results. Both the identified positions and their articulations, aligned with the analytical methodology, are recognised as discursive processes that significantly contribute to the emergence of the phenomenon under investigation [72].

## 3. Results

The responses collected through the protocols make it possible to analyse what people who use substances, also as citizens, think about the services offered by the TC through their activities.

How is the therapeutic community (TC) configured?

We can detect four different representations of the TC through discourse analysis Table 3).

### 3.1. The TC as a Refuge

The first representation we can consider is that of the TC as a refuge. Within this category of responses, the TC is seen as a space of welcoming and sustenance, shelter, and containment, and only afterwards can it take on a role referring to objectives related to therapeutic changes.

One participant affirms that *‘If you are addicted and lonely, many times you don’t know where to hit your head, and you see yourself with no way out’* (P8).

From these premises, some participants narrate the entrance with a rhetoric constructed based on losing control, seeing the TC as an alternative to irreversible situations. *‘In recent times, drinking was a daily act, so, remembering my past, I decided to ask for help before sinking into the abyss...and here I am...’ (*P17*).* This request is based, as we can see, on generic references, such as the request for a harbouring place or the reference to the fear of sinking into the abyss.

Within this configuration, the TC is represented as a place to turn to: ‘*When a person is by now in the state of destroying himself physically and psychologically’* (P13), when no alternative possibilities are identified, *‘I could no longer live’* (P29).

P20 reports that ‘For the umpteenth time, I lost everything: my job, my girlfriend, my family, my dignity, my respect for myself. On top of all this, I was about to lose my life, I saved myself by a miracle’.

The answers underline the fact that the choice to enter the TC is linked to a contingent and momentary experience of discomfort rather than to the progressive development of a plan of change and that the choice is restricted to a generic request for assistance. A representation of the TC is based on the perceived need to distance oneself from a situation of difficulty (I couldn’t bear it anymore) without any reference to a need for personal change.

Defining themselves as unable to manage consumption and to distance themselves from the contexts that facilitate it, several participants portray themselves as in need of generic help based on the exigencies of physical containment, symbolic and physical dislocation from the drugs, and support regarding problems such as loss of work, loss of housing, loss of emotional and family relationships, or health problems. P1 says that the ‘*TC is recommended for those who, after several attempts, have failed to stop permanent use, even more so if they struggle to break ties with old acquaintances and are unable to manage to rebuild their lives from a new beginning’*.

The institutionalised and residential nature of the TC is considered functional in detaching it from a context deemed uncontrollable, particularly due to issues of social exclusion, economic difficulties, or the inability to modulate consumption. *‘A help that I find fundamental already comes from the environment itself. Getting away from home, work, and the stress of everyday life is very much helping me to focus on myself and allows me, on the one hand, not to feel the weight of all these things, but at the same time to be able to concentrate on my own in a safe place’* (P11).

One participant reports that *‘The community is useful when you would like to stop but you can’t, and instead, you continue in the same direction, the substance is totally in control of your willpower’* (P17), while another says that ‘*When you see a person or a friend who is in a state of social dis-ease, living in absolute degradation, using and abusing, living a life in disarray...I would recommend going to a community to give meaning to life*’ (P19).

There is also mention of the possibility, through entrance into the TC, of distancing oneself from the constraints of a stereotyped and stigmatised identity: *‘Now I hope people will evaluate me for what I am... but I doubt it; there is so much ignorance in the world’ (P4). ‘It is not easy to accept being labelled by society... because, let’s not fool ourselves, people look at you differently’ (P3).*

Within this configuration, the community represents a safe refuge for the ‘drug addict’ who, in the very need of this refuge, feels the need not to be stigmatised.

### 3.2. The TC as the Final Liberation

A second category of responses configures the TC as the final liberation. Within this configuration, the consumption of psychotropic substances is represented as an addiction, maybe not explicitly a ‘disease’, from which one must be freed permanently. 

One respondent says that ‘After ten years of continuous use and after becoming a father, I decided to try this path to finally get rid of the addiction...to definitely stop use’ (P29), and similarly, another respondent affirms that ‘If a person uses, it is right that at some point in his life, he decides to get treatment and get definitively out of this tunnel’ (P14).

The TC is thus configured as a place for ceasing consumption, with abstinence becoming a primary objective. Abstinence assumes the role of a unique and definitive reference for change. The goal of abstinence is considered not pursuable independently; the theory ‘I can’t stop’ prevails. Therefore, the necessary contribution to the transformation is delegated to third parties: *‘When you want to quit on your own, it is almost never possible to do so... entering a path with addiction experts to find myself and realise that there are better things to do’ (*P6*).* Thus, the main index of liberation is the achievement of abstinence, configuring the treatment in terms of ‘momentary success’ (abstinence maintained to date) or ‘failure’ (stopping abstinence).

Some problems are objectified, reified, and inscribed in aut-aut dynamics that do not anticipate space for mediation. Some participants use the expression ‘I’ve decided to enter the community to get out of it’ (P26); another says that *‘I said stop to drugs; now I have to get out of it, to get rid of them’ (*P36*).* Service is thus represented as resolutive and salvific, aimed at people who say ‘*Enough*’.

Finally, among the answers emerges an objectification of ‘addiction’, considered an ontologically determined reality. The object of treatment is therefore not the person in his or her entirety, but only certain circumscribed aspects linked to consumption, conceiving the person as subordinate to his or her addiction: *‘You find yourself no longer in control of your life, and you find yourself at the mercy of a substance in the middle of an addiction’ (*P48*).*

### 3.3. The TC as a Place for the Acquisition of New Values

A third category of answers configures the TC as a place for acquiring new values, a configuration within which the dimension of moral judgement regarding past misconduct and the search for new values that can represent a reference for the future are central. Service is thus described as an educational and maturing place where people can understand themselves and learn other (‘healthy’ or ‘normal’) values to pivot a new life.

In this regard, one participant says that ‘I have to understand the mistakes I have made in life, not to make them in the future’ (P9). The abandonment of a ‘negative or wrong mentality’ is wished for (P24), affirming that ‘My goal is to change my mentality, my outlook on life, and my lifestyle’ (P6).

Entering the TC becomes a potential existential reset. One participant speaks of it as *‘a painful decision to enter the road or the path that can map out who I am and who I want to be’ (P3)*, reiterating the idea that the TC can generate a concrete shift in one’s biography, in deep and identity aspects, and thus hold it responsible for a radical axiological transformation.

Contextually, many participants consider the community a place for the acquisition of new values, and they use modes of confrontation and comparison between the present and the past. The past is narrated through theories regarding vice and moral corruption, reiterating a mortification and dramatisation of themselves and their biography. Abusive conduct is represented as detrimental to a ‘true identity’; in the TC, they search for the re-emergence of an authentic self.

In this regard, one respondent affirms that he needs to *‘understand who we are beyond use’* (P42), while another participant claims to want ‘to understand what led to the use of drugs’ (P22). Another participant reports that *‘I couldn’t understand who I became anymore...seeing the years go by and remain inside the vicious circle I had created for myself...’* (P8).

In this configuration, consumption is often used as the cause of a ruinous drift, not the conditions/actions that contributed to generating it and making it critical. If *‘addiction makes you deaf by taking possession of you’ (P2)*, one participant affirms that *‘The goal is to return to be the person I used to be, honest, sincere, loyal’ (P46)*, and another says that *‘I hope to go back to the guy I used to be before I was addicted’ (P55)*. Within this configuration, if we remove the ‘addiction’, we have the chance to go back to what we were before, without considering that this option also implies a return to the conditions in which consumption began and the consequences that today trigger the demand for change.

The generated position is that of an aspiration—and therefore a prior acceptance—for a change that is expressed through the ‘reflection’ and the understanding of specific consumption-related aspects: *‘I hope to take away this damn vice, and at the end of this path, I will start to cultivate new goals’ (*P49*).* One participant hopes to *‘return to living a healthy life without substance by understanding what leads me to always make the same mistakes, slip-ups, etc.’* (P11), emphasising the dimension of returning to a previous life that was subsequently spoiled by substance use.

### 3.4. The TC as a Symbol of Redemption towards Familiar Figures

For a proportion of newcomers, the abandonment by significant figures in their lives, particularly the family, is considered the main evidence of existential failure. Entry into the TC is, therefore, reported as an extreme attempt at reconnection, reconquest, and social redemption. One participant says that *‘I want to prove to myself and others that I have really cleaned myself up truly’ (*P41*).* Another person affirms that *‘The thing that made me decide was the fact that I was hurting myself and all the people who love me. I felt lonely; I had lost the will to live, and my only goal was to get through the day’ (*P38*).*

Another participant reports that ‘Since drugs marginalised me from everything and everyone, the desire to enter TC came from the need for social redemption to show people that I have changed and thus have a decent job and, above all, the satisfaction of the people who love me’ (P54). The choice to approach the service is spoken of as following a dynamic of encouragement or actual persuasion. This is sometimes explicit, as reported by one respondent, *‘I was driven by the continuous perseverance of people close to me because most probably if they had not been there now, I would still be on the street getting high’ (P37)*, while in other cases, it manifests itself with the loss of recognition and spatial–emotional proximity to family members.

The identity of an ‘addict’ is strategically assumed, showing awareness of one’s own discomfort and willingness to ask for help, to the point of sticking to the identity of a ‘sick person’. This process seems consistent with the attempt to show oneself as changed, eventually determined to take responsibility and face problems. Entry into the TC is thus charged with a high symbolic value, representing in and of itself a change already in progress. The prevailing instance is providing evidence of the change, demonstrating it to themselves and others.

## 4. Discussion

As discussed above, for some participants, the value of the service lies solely in its ability to provide sustenance and relief from acute distress. While this is sometimes seen as functional in creating a safe space to initiate pathways of help, what emerges from the participants’ narratives is the absence of explicit planning. Entry into a community is described as a purely biographical coincidence, supported by a passive welfare-seeking issue. Frank and Walters [34] also noted that entry into TCs is often the result of an interactive and social process rather than an individual decision stemming from a reflective and introspective journey.

Peer and family pressure, along with aspects of criminalisation and the stigmatisation of consumption, appear central to promoting the decision to seek a service. The self-definitions collected through the research protocols highlight how participants tend to dramatise their condition and adopt a self-victimising rhetoric. Often, after the initial feelings of well-being due to abstinence from use (*‘I have stopped using here’*, P12), individuals who use substances tend to attribute responsibility for their behaviour to ‘drug addiction’, thereby depriving themselves of agency, medicalising their situation, and delegating their actions to specialists.

Another noteworthy aspect is how TCs assume the character of a ‘Hail Mary pass’, permeated by an implicit institutional resignation [32], with the evaluation of their efficacy often reduced to their competence in facilitating abstinence. This seems to result from multiple processes linked to socially available discourses regarding consumption, previous failed experiences of abstinence, attempts to construct a non-dependent identity, and the moral order within which these discourses are generated [32,65]. Within this framework, the call to action for TCs is configured along the bipolarity of being powerless–responsible, such that it becomes the result of contact with an inherently uncontrollable substance or reckless and deliberate conduct, as also reported by Harré and Van Langenhove [69].

The theory of ‘substance use as an organic disease’ and the simultaneous exclusive search for a ‘pharmacological cure’ trigger a dichotomous logic based on healthy/sick and, consequently, cured/not cured individuals. One implication of this is to reify addiction as a circumscribed pathological core. The second is to consider this core as detached from the individual using the substance and their history, as also argued by Tibirica et al. [73], who pointed out this harmful dichotomy.

The aim (sometimes implicit and sometimes explicit) of the request for intervention is the interruption of substance consumption, thus triggering the reference to abstinence as the sole criterion. This assumption implies that a possible ‘relapse’ is considered a failure of the programme, dramatising the problem and leading the individual to justify substance use again. Consequently, contact with the substance triggers the resumption of use, diminishing the role of personal choice and responsibility concerning the initial contact. If substance consumption is the issue to be avoided, the opportunity to articulate one’s hesitations about quitting becomes critical. This leads to the paradox whereby, in the TC, the issue of substance consumption becomes silenced, with consumers utilising discourses of sobriety as mere adaptation and pursuit of secondary goals.

Despite its evolution into a service with less binding access thresholds, TCs remain pathways that imply a break with previous behaviours and contexts. This is perceived by participants as both an added value and a significant sacrifice. The nature of the (almost) total institution is assessed as necessary and welcome to generate that biographical shock useful for starting anew, with the TC experience serving as a junction between a before and an after.

If the protected and isolated nature of the TC can be salvific in moments of extreme crisis, it is essential to consider the capacity to construct other identities within a service that binds individuals to a separate context, both spatially and symbolically. This contradicts the principles of community care [74].

We have seen how, for participants, the choice to enter the TC often represents ‘the last resort’. It is only considered in the face of ‘losing everything’ or ‘hitting *rock bottom’ (‘if the person does not touch bottom, it is like dealing with walls’, P30)*, prompting questions about the type of consumers the service can intercept, the prevention practices in action, and how the TC presents itself to those it might benefit [75].

A further implication of the detected service configurations is that entry into TCs is used strategically for communicative purposes, attempting to promote in the eyes of others (and thus in their own) evidence of change aimed at secondary goals. This offers a potentially generative ground to establish some desirable repercussions of a life away from substances. At the same time, it entails the need to quickly encourage individuals to co-generate a project that, otherwise, risks being reduced to the objective of mere permanence in the service.

Moreover, individuals are relational beings who need to be considered to avoid implementing interventions detached from the real contexts they inhabit, reflecting on the sense of identity that substance consumption has socially. Rather than being ‘for others’, overshadowed by the substances, entry seems functional to reconfigure oneself ‘in the eyes of others’, alleviating feelings of guilt, inadequacy, and the concrete and material repercussions dictated by disavowal.

Overall, TCs are described as distant from the rest of the world, resulting from a symbolic gap that delineates an inside and an outside. Starting from this assumption, it is even more pertinent to consider which identity configurations are being generated. Which aspects of the newcomer’s self cross the threshold of the service, and which become the focus of attention? If the TC is a space apart from the world, what possibilities does it have of generating changes that resonate beyond its confines?

## 5. Conclusions

Based on the texts collected, it was possible to detect how TCs accommodated discomforts that extended beyond a clinical and specialist domain, as substance consumption—at least that intercepted by the services—is often intertwined with critical issues of economic, work, social, and relational aspects. This underscores the need to develop reflection on the role of TCs, both present and future, within a broad and interconnected system of services [21].

Overall, participants positioned themselves as passive recipients of treatment decided by others, and their approach to the service appears to respond to contextual, and often emergency, needs, rather than personal projects developed over time. This demand is predominantly based on the consequences of consumption and calls for both causes and consequences to be eliminated.

Intervention focused solely on consumption or achieving abstinence cannot be considered exhaustive and risks validating the notion that the problem lies with the substance or the implications concerning abstinence such as diminished feelings of responsibility for use and a loss of agency. Building on this research, we consider it useful to develop tools and methodologies not aimed at confirming this belief but rather favouring the individual’s ownership of their path based on requests and questions defined in intentional terms. Thus, access to the service is a decisive event with high symbolic and pragmatic value, requiring accompaniment and support to co-construct the meaning of the pathway and its function in the individual’s life within a framework of co-responsibility.

The shared construction of goals and pathways in the community can be a potentially innovative first step for individuals entering the community. This approach markedly differs from the passive, substance-dependent, and service-dependent attitudes observed in participants during our research. Involvement can trigger a sense of responsibility towards one’s own path and health care [76] and encourage the activation of social inclusion processes, as confirmed by other studies [51], which showed that storytelling in drug treatment is an interactive achievement.

Finally, it can foster the legitimisation of treatment as a shared and personalised pathway, thereby increasing the likelihood of a positive outcome. This was noted by other authors [10,28] who emphasised the importance of involving substance users in services and the necessity of reducing barriers to involvement.

In this way, users will be better equipped to anticipate the challenges they may encounter during the process, facilitating their management from the outset. The shared construction requires and simultaneously generates a reduction in the rigidity of the relationship between substance users and community workers (healthcare professionals providing treatment at the facility), positively transforming the helping relationship.

Regarding the limitations of this study, the written form of the answers presented some challenges. First, we encountered difficulty in writing the texts for users (a minority) with minimal literacy levels. Since this remains a reality in TCs, it is important to include elements of flexibility that can simultaneously promote the sharing of significant elements while maintaining methodological consistency.

There were also several responses marked with a peremptory ‘I don’t know’, which, while potentially useful from an operational perspective as indicative of how individuals legitimised the service and the requests received, could have been further explored to examine the underlying contents and processes.

## Figures and Tables

**Table 1:** Demographic data.

Gender	N	%
Male	41	71.9
Female	16	28.1
**Age range**		
18–30	20	35.1
31–50	26	45.6
51–70	11	19.3
Age average	37.7	/
**Typology of entry**		
Regular entry	42	73.7
Alternative measure programme	15	26.3

**Table 2:** Research protocol.

Research Area: Discursive Configuration Regarding Entry into the Therapeutic Community
How did you come to the decision to enter a therapeutic community (TC)? What is your image of a therapeutic community? What goals do you intend to achieve in this process? How do you imagine a therapeutic community works?
5. How do you think the TC could facilitate you in achieving your goals? 6. What situations would you recommend to someone seeking help from a TC?

**Table 3:** Result diagram.

Results: How the Therapeutic Community (TC) Is Configured
The TC as a Refuge The TC as the Final Liberation The TC as a Place for the Acquisition of New Values The TC as a Symbol of Redemption towards Familiar Figures

## Data Availability

The dataset presented is not available for privacy reasons.
